# Effects of Intermittent Fasting on Brain Metabolism

**DOI:** 10.3390/nu14061275

**Published:** 2022-03-17

**Authors:** Alex Brocchi, Eleni Rebelos, Angela Dardano, Michele Mantuano, Giuseppe Daniele

**Affiliations:** 1 Department of Clinical and Experimental Medicine, University of Pisa, 56124 Pisa, Italy; alexbrocchi@gmail.com (A.B.); angela.dardano@unipi.it (A.D.); michelemantuano8@gmail.com (M.M.); 2 Institute of Clinical Physiology, National Research Council (CNR), 56124 Pisa, Italy; elenirebelos@gmail.com

**Keywords:** intermittent fasting, ketone bodies, neuroprotection

## Abstract

We are facing an obesity epidemic, and obesity itself and its close companion, type 2 diabetes, are independent risk factors for neurodegeneration. While most medical treatments fail to induce a clinically meaningful improvement in neurodegenerative disorders, lifestyle interventions have emerged in the spotlight. A recently rediscovered approach is intermittent fasting (IF), which, compared to the classic caloric restriction regimens, limits only the time of eating, rather than the number of calories allowed per day. There is already a large amount of evidence from preclinical and clinical studies showing the beneficial effects of IF. In this review, we specifically focus on the effects of IF on brain metabolism. Key molecular players modified during IF and involved in its beneficial central effects (ketone bodies, BDNF, GABA, GH/IGF-1, FGF2, sirtuin-3, mTOR, and gut microbiota) are identified and discussed. Studies suggest that IF induces several molecular and cellular adaptations in neurons, which, overall, enhance cellular stress resistance, synaptic plasticity, and neurogenesis. Still, the absence of guidelines regarding the application of IF to patients hampers its broad utilization in clinical practice, and further studies are needed to improve our knowledge on the different IF protocols and long-term effects of IF on brain metabolism before it can be widely prescribed.

## 1. Introduction

The brain is a dynamic and plastic organ whose complex activity is accompanied by an enormous consumption of energy. This wide energy demand is necessary to maintain processes such as transmembrane potential preservation and action potential generation, fundamental for neuronal signaling, the main task of the cells of the central nervous system (CNS). Other not-negligible functions are represented by basic cellular activities also referred to as “housekeeping”: macromolecule and organelle turnover and axonal transportation, for example [1]. Despite this great demand, the brain does not have a sufficient reservoir of energy: only a limited amount of glycogen is stored in the CNS. It is therefore forced to rely on a constant supply of energy-rich substrates from the blood through the blood–brain barrier (BBB) [2]. Under physiological conditions, the brain mainly consumes glucose, but other substances can contribute to metabolism in different situations, for example when glucose availability is limited. These alternative fuels are: ketone bodies, lactate, and medium-chain triglycerides [3]. In an average-sized resting adult, fasting for about 10-14 h is sufficient to deplete the liver glycogen stores: this is the moment when the ketone bodies become protagonists. Beta-hydroxybutyrate (BHB) is the principal ketone body, produced in the liver mitochondria from the oxidation of adipose-tissue-derived fatty acids; its plasma concentrations can increase significantly, from baseline levels of 0.05 mM to 25 mM during ketogenic diet protocols or diabetic ketoacidosis [4].

Recent evidence shows that the metabolic changes resulting from a fasting state, may enhance brain function in terms of better cognitive performance, increased neuroplasticity, and resistance to injury and disease [5,6]. On the other hand, it is clear that overfeeding and metabolic diseases are harmful for brain metabolism and aggravate neurodegenerative disease manifestations. Therefore, given the lack of validated and effective therapies, the focus is now on modifiable lifestyle factors such as dietary habits. In particular, dietary restriction appears to be one of the most promising approaches and nowadays is a trending topic in scientific production [7,8]. Caloric restriction (CR) is one of the dietary regimens that have been demonstrated as exerting positive effects on cognition [9,10,11]. However, CR may be associated with malnourishment and lean mass loss and may be hardly acceptable, leading to low compliance [12,13]. Moreover, the body tends to get used to chronic CR, reducing basal energy expenditure even when adjusted for weight loss [14].

Therefore, nutritional habits cannot simply be reduced to the quantity and macromolecular quality of food eaten, but the frequency of meals, their time, and the duration of the interprandial fast are also important. Intermittent fasting (IF) is a concept of a dietary pattern in which eating time, and not the amount or composition of the food, is limited. There is a great variety of types of intermittent fasting regimens, but the most popular can be placed into one of these categories [15,16].

- Time-restricted eating (TRE): eating is restricted throughout the day to a limited number of hours (for example out of 24 h, 16 are devoted to fasting and 8 to eating). This intervention can be further divided into early (eTRE) and late time-restricted eating (lTRE).
- Alternate-day fasting (ADF): fasting days alternate with those of free eating in various schemes (one of the most popular is the 5/2 method: fasting for 2 non-consecutive days in a week and ad libitum eating in the other 5).
- Modified alternate-day fasting: similar to ADF but during fasting days a low-calorie intake is allowed (15–25% of the caloric needs).
- Other types of fasting, such as the one followed for religious or spiritual needs.

Therefore, IF is a recently rediscovered method that can provide further benefits over caloric restriction alone. In fact, a recent meta-analysis showed that compared to a control diet, IF leads to larger decline in BMI, fasting plasma glucose levels, and improvement in insulin sensitivity [17].

In this review, we analyze the wide body of literature available today to focus on: (1) the mechanisms induced by IF in neuronal cells; (2) the effects of IF on brain health, in both animal models and humans; (3) possible future clinical applications of IF in the field of neurological pathologies.

## 2. Materials and Methods

We comprehensively and carefully analyzed the available studies and reviews found in research on PubMed and Google Scholar with the following search items: fasting, intermittent fasting, time-restricted eating, alternate-day fasting, and modified fasting. We included studies relying on the following criteria: (1) controlled studies considering at least a 16 h fasting period; (2) papers where brain metabolism and function parameters were studied.

## 3. Results

### 3.1. Fasting and Brain Metabolism

The human brain represents ~2% of body weight but accounts for ~25% of the body’s resting metabolic rate [18]. Most of energy consumption is related to signaling, with the remainder used for essential cellular activities including turnover of proteins, nucleotides, phospholipids and axoplasmic transport. In conditions of normal food availability, glucose is the main energy substrate for the brain, and oxidation of carbohydrates accounts for almost the entire oxygen used. However, during conditions of food deprivation, energy sources for both body and brain are highly modulated and a sort of metabolic shift from glucose to ketones occurs [19,20]. Glucose and fatty acids are the main sources of energy used to perform and maintain cellular functions in tissues and organs in most mammals. After meals, glucose is used to obtain energy and, if in excess, is stored in the form of glycogen in the liver, which represents the main reservoir. Fatty acids are stored in adipocytes as triglycerides. In humans, during fasting, liver glycogen stores are disassembled to produce glucose via glycogenolysis; depending on the amount of glycogen hepatic reserve and physical activity, 12 to 24 h of food deprivation result in a complete depletion of hepatic glycogen. On the other hand, lipolysis of triacylglycerols in adipose tissue generates free fatty acids (FFAs), which are released into the bloodstream and then transported into hepatocytes, where they are metabolized via β-oxidation to acetylCoA, which is used to sustain the production of ketones such as β-hydroxybutyrate (BHB) and acetoacetate. Therefore, in fasting conditions, a metabolic switch occurs, as the body shifts from utilization of glucose from glycogenolysis to fatty acids and fatty acid-derived ketones [21]. Ketones pass the blood–brain barrier and enter neurons via mocarboxylic acid transporters (MCTs) localized on the membranes of vascular endothelial cells and neurons. Eight different members of the MCT transporter family have been identified, with the MCT1 and -2 transporters being the most thoroughly characterized in the adult brain: MCT1 is localized on vascular endothelium cells and astrocytes, while the higher-affinity MCT2 is localized primarily on neurons [22]. Adult brain neurons express all of the enzymes necessary for using ketones in order to produce energy when present in blood at high levels (D-β-hydroxybutyrate dehydrogenase, acetoacetate-succinyl-CoA transferase and acetoacetyl CoA-thiolase). Here, they are metabolized in a process termed ketolysis; ketones are converted into acetyl coenzyme A, which enters the tricarboxylic acid cycle in mitochondria in order to produce adenosine triphosphate (ATP). Nonetheless, during prolonged starvation, ketone bodies can provide about half of the oxidative fuel for the human brain, with glucose accounting for the remainder [23]. A high-fat, low-carb diet increases the concentration of ketones in the blood and reduces glucose utilization proportionately in the rat brain [24], and when ketones are infused into human subjects, their fractional oxidation rate in neurons and astrocytes is similar to that of glucose [25]. A notable finding is that acute intravenous infusion of BHB, at such a rate as to saturate transport protein binding capacity (~17 mmol/L), displaced oxidation of glucose in pentobarbital-anesthetized rats with isoelectric electroencephalograph, indicating that BHB can fully support non-signaling activities [26,27]. However, when infused into conscious resting rats, BHB accounted for only ~50% of the energy required by signaling, in equilibrium with glucose. To summarize, brain activity, metabolic demand, and blood flow rate are very closely linked, so neuronal activation raises local demand for ATP, stimulates ATP production, and increases blood flow to the excited area. Alternative oxidative substrates can potentially make up for a considerable fraction of the energy required by the adult brain in vivo, however, they fail to satisfy the whole demand due to transport protein capacity saturation and due to inability to wholly replace glycolysis, which consistent with failure of lactate, pyruvate, or BHB to prevent or reverse effects of hypoglycemia [28]. Moreover, mitochondrial metabolism of BHB does not involve the malate-aspartate shuttle, which is extremely important to transfer reducing equivalents (NADH) from cytosol to mitochondria and maintain an efficient glycolysis [29].

### 3.2. Molecular, Hormonal, and Systemic Mechanisms by Which IF Affects Neural Function

In fasting conditions, neurons are involved in several molecular and cellular adaptations. During the transition from glucose to ketone bodies as the main cellular source of energy, multiple signaling pathways are induced in neurons in response to the metabolic switch, enhancing cellular stress resistance, synaptic plasticity, and neurogenesis.

#### 3.2.1. Brain-Derived Neurotrophic Factor (BDNF)

BDNF, a member of the nerve growth factor family, is of paramount importance in maintaining neuronal survival, synaptic function, hippocampal neurogenesis, learning, and memory, as well as a major regulator of glucose metabolism and body [30]. Robust evidence supports the enhanced production of brain-derived neurotrophic factor (BDNF) as one of the most important neuronal adaptations to IF [31]. In fact, the metabolic switch that occurs during food deprivation stimulates the excitatory synaptic activity in neurons, triggering calcium influx through membrane channels and resulting in the activation of multiple kinases and signaling pathways that induce the expression of different genes that ultimately encode proteins involved in cellular stress adaptation, one of which is BDNF [32]. As demonstrated by two studies, BHB induces *Bdnf* gene expression in hippocampal and cortical neurons in cell culture and in vivo [33,34]. Two mechanisms by which BHB enhances BDNF production have been described: first, it may upregulate *Bdnf* gene transcription by inhibiting histone deacetylase, which normally represses *Bdnf* expression [34]; secondly, BHB induces the transcription of nuclear factor κB (NF-κB) in neurons, which in turn upregulates *Bdnf* expression [33]. These results demonstrate that BHB, which is typically elevated during fasting, not only represents itself a signal for neurons that the metabolic switch has occurred, but is also a peripheral signal that triggers signaling pathways that enhance neuronal stress resistance and neuroplasticity. A study by Duan et al. conducted on *Bdnf* heterozygous knockout (*Bdnf* +/−) mice demonstrated that alternate-day fasting regimen for three months reversed several abnormal phenotypes including obesity and hyperphagia and increased locomotor activity [35]. IF increased BDNF brain levels in BDNF-deficient mice up to the level in wild-type mice fed ad libitum and reduced the levels of circulating glucose and insulin so much to normalize glucose tolerance and insulin tolerance tests [35]. In two other rodent studies, IF triggered the expression of BDNF, inducing an increased density of dendritic spines in hippocampal dentate granule neurons, enhancing memory and spatial learning [36], and ultimately potentiating hippocampal neurogenesis [37].

Recently, it has been shown that BDNF can reduce neuroinflammation [38], which is an important determinant in neurodegenerative diseases [39]. In addition, evidence for reduced neuroinflammation following a fasting regimen has also been provided. In ischemic brain injury mice models, lower levels of TNFα and IL-6 were reported in the cortex and striatum of mice maintained on an IF regimen compared to mice on an ad libitum diet. Moreover, intermittent fasting significantly reduced ischemia-induced increases in TNFα and IL-6 levels [40]. Similarly, alternate-day fasting regimen reduced oxidative stress markers in the hippocampi of rat models of chronic cerebral hypoperfusion, compared to ad libitum fed rats [41].

#### 3.2.2. Transcription Factor Peroxisome Proliferator-Activated Receptor γ Coactivator 1α (PGC1α)

PGC1α is a transcriptional regulator of mitochondrial biogenesis that plays a pivotal role in regulating hippocampal synaptogenesis [32], as well as in the long term maintenance of dendritic spines of hippocampal dentate granule neurons, as reported in a study conducted on mice [42]. It acts by upregulating oxidative phosphorylation, mitochondrial DNA transcription, and mitochondrial protein import [32]. All these effects contribute to increasing the number of mitochondria that ultimately can support the function of new and potentiated synapses. As recently reported by Liu et al. [43], a 28-day IF regimen in diabetic mice led to a significant increase inPGC1α expression and mitochondrial biogenesis; this was associated with an improvement in spatial memory and in cognitive function, measured with the Morris water-maze test. Interestingly, PGC1α can also enhance *Bdnf* expression [44].

#### 3.2.3. SIRT3

Sirtuin 3 (SIRT3) is a NAD+-dependent deacetylase that affects many facets of mitochondrial biology through the regulation of vast networks of metabolic and non-metabolic enzymes, ensuring mitochondrial homeostasis during stress conditions [45]. It is critical for fatty acid oxidation and ketogenesis during fasting. Loss-of-function studies on mouse models have linked impaired sirtuin function to age-related disease development, including neurodegeneration [46]. Supplementation of NAD+ improves health and restores mitochondrial homeostasis in model systems [45]. Similarly, SIRT3 overexpression has been found to be neuroprotective [46]. Traba and colleagues studied wild-type and *Sirt3* knock-out mice and showed that after a prolonged period of fasting, only in wild-type mice was a suppression of pro-inflammatory IL-1 β secretion evident, whereas this fasting-mediated effect was absent in *Sirt3* KO mice (+/+). The negative stimulatory effect of *Sirt3* on the NLRP3 inflammasome occurred via activation of superoxide dismutase 2 (SOD2) [47]. Moreover, *Sirt3* gene expression was relatively downregulated after Ramadan IF in patients with obesity compared to non-obese controls [48]. Since the expression of sirtuins is enhanced by inflammation, the authors suggested that the reported decrease in *Sirt3* expression could be attributed to a modulation of the inflammatory and anti-inflammatory markers following Ramadan IF.

#### 3.2.4. mTOR and Autophagy

The mammalian target of rapamycin is a kinase involved in processes that regulate cellular protein synthesis in response to fluctuations in the availability of glucose and amino acids. The availability of nutrients switches on mTOR activity, enhancing protein and lipid synthesis and ultimately setting the cell into a sort of “growth mode” [32]. On the contrary, during food deprivation, mTOR is switched off and autophagy is stimulated. Autophagy is a cellular process mediated by the lysosomal system, which regulates the clearance of damaged proteins and organelles and contributes to cellular membrane turnover and vescicular transport. By counteracting the accumulation of damaged or misfolded proteins, as it happens in most of neurodegenerative diseases, the upregulation of the autophagy process in response to fasting can potentially exert a protective role in neurodegenerative diseases. On the contrary, the downregulation of the lysosome pathway can increase the risk of developing neurodegenerative diseases, as described in the study by Hara et al. [49], where *Atg5* (autophagy-related 5) gene-deficient mice developed progressive deficits in motor function, mirrored by deposition of cytoplasmic inclusion bodies and aggregates in neurons. Given these considerations, the potential role of the autophagy-lysosomal pathway has been studied also in an Alzheimer’s disease mouse model [50]. Although fasted mice showed an increase in number, size, and signal intensity of autophagosomes in neurons, the activated autophagy was insufficient to degrade the intracellular beta amyloid, which was increased by the enhanced uptake from extracellular space after fasting. Interestingly, age-specific responses to mTOR inhibition following IF on zebrafish have been reported [51]. An activated mTOR pathway may be involved in several neurologic disorders, including epilepsy [52], autistic spectrum disorders [53], multiple sclerosis [54,55], and Parkinson’s disease [56,57]. Thus, inhibiting mTOR through dietary modification (i.e., IF) seems a promising prospect for these neurologic disorders. However, current data are insufficient and further studies are necessary to clarify the effects of fasting-induced autophagy on protecting from neurodegeneration.

#### 3.2.5. FGF2

Fibroblast growth factor 2 (FGF2) belongs to the family of fibroblast growth factors and is a wide-spectrum mitogenic, angiogenic, and neurotrophic factor expressed at low levels in many tissues and cell but at high concentrations in CNS [58]. FGF2 has been implicated in a multitude of physiological processes, such as stimulating neural stem cell proliferation and cell survival during brain development [59] and protecting neurons against oxidative stress [60]. Furthermore, a significant reduction in neuronal density has been reported in most layers of the motor cortex in FGF2−/− mice (57). As reported in the study of Arumugam et al., mice kept on an IF diet for 4–5 months prior to experimental stroke had lower rates of and brain damage and exhibited increased levels of FGF2 in the cortex and striatum compared with those fed ad libitum. Perhaps, food-deprivation-mediated FGF2 upregulation may reduce brain damage and improve functional outcome in stroke model, protecting neural cells and enhancing synaptic plasticity [40].

#### 3.2.6. Gamma-Aminobutyric Acid (GABA)

GABA is the main excitatory neurotransmitter in the mammalian brain and is involved in the control of excitability, information processing, synchronization of neuronal activity, neuroplasticity, and processes of learning and memory [61]. Moreover, GABA regulates the response of neural circuits to environmental challenges, activating pathways that mediate structural and functional modifications, such as synaptogenesis, long-term potentiation, and long-term depression, which are of paramount importance in neuroplasticity. By binding to its receptor, GABA activates a downstream pathway that converges on some transcription factors including cAMP-responsive element-binding protein (CREB) and NF-κB, which, in turn, induce the expression of many different genes that encode proteins involved in cellular stress adaptation, one of which is BDNF [32]. Ketones produced during food deprivation can upregulate GABA, and, by doing so, may contribute to mediate the adaptive responses of neural cells to fasting.

#### 3.2.7. Ghrelin

Ghrelin is a hormone produced by the P/D1 cells located at the bottom of the human stomach and by the epsilon cells of the pancreas in response to fasting. By binding to receptors located in the arcuate nucleus, it exerts its orexigenic effect; moreover, in the CNS, ghrelin affects neuroplasticity, reduces cell death, and increases neuronal survival [62], improving test results for the assessment of attention and executive functions [63]. Furthermore, ghrelin stimulates serotonergic neurons that innervate hippocampus, enhancing learning and memory [64].

#### 3.2.8. GH e IGF-I

Growth hormone (GH) or somatotropin is a peptide hormone produced by somatotropic cells of the anterior pituitary gland that acts as an anabolic agent, stimulating growth, cell reproduction, and cell regeneration. Its primary function is the promotion of linear growth during youth, and most of the effects are mediated by insulin-like growth factor 1 (IGF-1), produced in the liver. GH, via IGF-1, increases protein synthesis by enhancing amino acid uptake and directly triggering the transcription and translation of mRNA. In addition, GH tends to reduce protein catabolism by mobilizing fat as a more efficient energy source: the release of fatty acids from adipose tissue and their conversion to acetyl-CoA provides energy to cells and elicits a protein-sparing effect that contributes to promote growth and development. Moreover, GH exerts neuroprotective effects [65], and it also appears to improve cognitive function, learning, and memory in patients with GH-deficiency-related cognitive impairment [66]. GH secretion of GH is mainly stimulated by growth hormone releasing hormone (GHRH), while somatostatin is the most important inhibitor. Among the factors that enhance GH secretion, the role of fasting and ghrelin has been well-documented [67]. In a review comparing IF and caloric restriction, it was found that weight loss observed during IF was associated with a greater maintenance of lean mass, and GH has been proposed as one of the factors that could explain it, as it may be secreted to a greater extent in conditions of prolonged food deprivation rather than in a caloric restriction approach [68]. Moreover, IGF1, acts as a neurotrophic factor due to its capacity to enhance neuroplasticity and protecting neurons against metabolic and oxidative stress [69]. As reported by Llorens-Martin et al., IGF1 signaling is upregulated in response to fasting [70], although circulating IGF1 levels were demonstrated to be reduced in mice on a 4:3 diet [71]. One possible explanation is that lower levels of IGF1 may be consequence of a negative energy balance due to IF regimen; however, it is possible that fasting increases the IGF1 receptor sensitivity, enhancing the net effects of the hormone [32].

#### 3.2.9. Gut Microbiota

Gut microbiota has a critical role in regulating brain energy homeostasis and synaptic transmission [72], and by these mechanisms it can modulate cognitive functions. In order to examine the impact of gut microbiota on cognitive function during IF, Liu et al. [43] conducted a study on type 2 diabetes rodent models with cognitive impairment. Three-month-old db/db mice were fed either ad libitum diet or fasted at 24 h intervals for 28 days. IF treatment increased insulin sensitivity (as demonstrated by lower values of HOMA-IR), stimulated the hippocampal insulin signaling pathway, enhanced the expression of *Bdnf*, promoted mitochondrial biogenesis in hippocampus and improved cognitive deficits and spatial memory (as evidenced from a Morris water-maze test) of db/db mice. IF led to a restructuration of gut microbiota and alterations in the microbial metabolites with increased levels of bile acids, that have already been reported to have a strong association with cognitive functions [73]. Intriguingly, the beneficial effects of IF on cognitive function were suppressed after removing the gut microbiota by antibiotics treatment, suggesting a key role of microbiota composition and its derived metabolites in mediating IF-induced neuronal effects.

**Figure 1:**
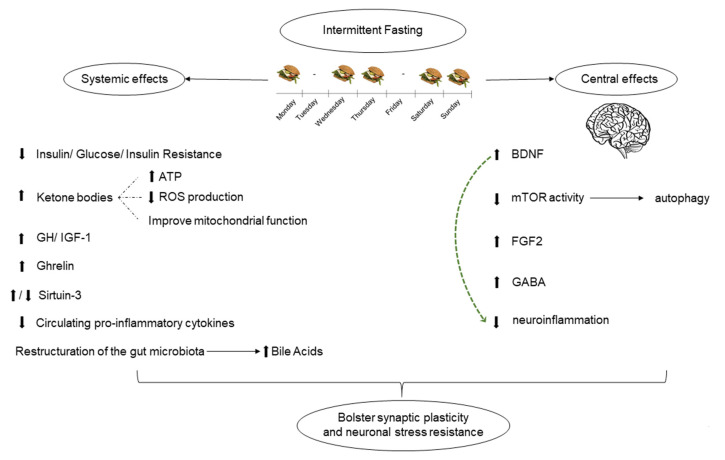
Summarizes the systemic and central effects of intermittent fasting, which ultimately confer neuroprotection. arrows pointing up, increased levels; arrows pointing down, decreased levels.

In summary, available data suggest that IF induces signaling pathways in neurons and in other tissues that, taken together, bolster synaptic plasticity and neuronal stress resistance (Figure 1).

### 3.3. Role of Obesity and Insulin-Resistance on Cognitive Impairment and Effects of Intermittent Fasting

Obesity has been linked to several structural and functional alterations in CNS, including, among others, altered brain substrate uptake [74,75], reduced volume of the hippocampus [76], and hyperintensity areas in the white matter [76]. Obesity also represents an independent risk factor for Alzheimer’s disease, and a recent meta-analysis showed that midlife obesity increases the risk of dementia and Alzheimer’s disease compared to late life obesity [77].

From a mechanistic standpoint, chronic low-grade inflammation, central insulin resistance, and decreased CNS insulin levels [78,79] have all been implicated in the increased risk for neurodegeneration in patients with obesity. Imbalanced diets, such as the Western Diet (WD), contribute to increasing this risk. WD is based on high-calorie, ultra-processed foods (rich in simple carbohydrates, saturated fatty acids, cholesterol, and salt and poor in fibers and mono- and poly-unsaturated fatty acids) and can lead to obesity. The molecular mechanisms through which WD can predispose individuals to neurodegeneration have been extensively reviewed elsewhere [80], but in essence, evidence suggests that WD promotes neuroinflammation through an acceleration of brain amyloid deposition and p-tau pathology [80].

Given the proven association between obesity, insulin resistance, and the development of cognitive impairment and some forms of dementia, strategies aimed at reducing weight excess may also lead to beneficial results in terms of reducing the burden of neurological manifestations. Indeed, several studies have shown that weight loss via caloric restriction leads to improvement in verbal and working memory, language, executive functions, and global cognition [10,81].

Preclinical evidences regarding the effect of IF on cognitive function are summarized in Table 1; studies on the effects of IF on cognition in humans are presented in Section 3.4. Studies on rodents have shown that an IF regimen lasting 6 to 8 months significantly enhanced spatial learning and memory compared to controls fed daily [82]. Similarly, in a transgenic mouse model of Alzheimer’s disease, 1 year of a 40% CR or IF approach ameliorated spatial memory acquisition and retention in the Morris water-maze test [83]. Furthermore, spatial memory, associative memory, and working memory have all been shown to be enhanced in mice fed according to an IF regimen as reported by Wahl et al. [84].

### 3.4. Possible Clinical Applications of Intermittent Fasting in Neurological Disorders

To date, it is not clear whether the beneficial effects of IF on CNS health observed in cellular and animal experimental models are also valid in a clinical context. Having an answer to this question would be useful, since validated and effective therapies are not yet available for many disorders affecting the CNS, in particular neurodegenerative diseases. For this purpose, the neuroprotective effects of IF could represent a valid care option, especially in a prevention program or in the earliest stages of the disease.

Alzheimer Disease (AD) is the most frequent neurodegenerative disease, predominantly affecting people over 70 years old [91]. The dramatic age-related increase in neurodegenerative disease prevalence is a current worrying public health problem given the rise in the average age of the population and the increase in the number of people in the highest age groups, which are consequences of the improvement in global living conditions in recent decades [92]. A very interesting fact is that more than 80% of patients with AD have T2DM or altered fasting blood glucose levels, demonstrating an important connection between dysmetabolic conditions and neurodegenerative diseases [93].

A recent longitudinal study assessed the effects of IF on cognitive function among lean elderly subjects with mild cognitive impairment (MCI), stratified for IF compliance. After 36 months of IF (TRE from sunrise until sunset), subjects in the regular IF group achieved better cognitive scores in most tests assessed (MMSE, digit span, RAVLT, MoCA, and Digit symbol) and improved their cognitive function compared with the less IF-compliant groups. Furthermore, in the regular IF group, improvements in adiposity measures, as well as in HDL-cholesterol, basal insulin, CRP, and blood glucose levels, were noted [87].

In another recent pilot study, the effects of a time-restricted feeding (TRF) were evaluated on a small cohort of overweight, sedentary, over 65 years old adults with a mean basal blood glucose in the range of IFG. TRF was designed to contemplate a 16 h a day fasting with no dietary restrictions during the other 8 h. Although statistical significance was not reached, researchers reported an improvement in health-related quality of life score but not in cognitive function after a 4-week TRF intervention. A good adherence was obtained across the participants, with no severe adverse events occurring, and, on average, a 2.6 kg weight loss was achieved, in line with a previous TRF intervention study. As stated by the same authors, the short duration, and the small cohort of the study limit the generalizability of the results registered [88].

Observational studies have also demonstrated the beneficial effects of IF on cognition. In older Italian adults, subjects with an eating time window duration of more than 10 h were compared to those with an eating time restricted to less than 10 h. After adjustment for confounders, subjects on IF had a lower risk for cognitive impairment (as assessed by the Short Portable Mental Status Questionnaire) [89]. In the same line, in a large cohort of adults (*N* = 1572), it was observed, after adjusting for confounders, that subjects older than 70 years old who were already practicing IF as their habitual eating pattern (eating within 8 h or less) had a decreased risk of mental health distress [90].

The available studies assessing the effects of IF on cognitive function are summarized in Table 1. Although further studies of IF and cognition are needed, this dietary approach seems to be promising in ameliorating cognitive function while also being a viable and safe dietary alternative in older adults, a population at a high risk for malnourishment and neurodegenerative diseases.

Several studies assessed the effect of Ramadan IF on cognition, yielding mixed results, as reviewed by Qasrawi et al. [94]. These studies, however, may be plagued by the deleterious effect of late heavy eating on sleeping patterns, which can impair the physiological circadian rhythm [95] and lead to a reduction in the effects of a prolonged fasting. In one well-controlled but small study in young and healthy subjects, BaHammam and colleagues reported no effects of Ramadan on cognition [86].

Data from clinical trials about the effects of IF on other neurological affections are lacking, but evidence from the experimental field suggest that fasting or physical activity induce intermittent metabolic switch and may exert a beneficial effect on mood and anxiety, ameliorating symptoms of depression. In this regard, *Bdnf* expression upregulation in hippocampal and cortical neurons may play an important role, and this mechanism may be shared between IF and physical activity, so a synergic effect should be investigated in future protocols [32,34]. During the last 30 years, the incidence of autism spectrum disorders (ASD), which is characterized by repetitive behaviors and communication deficits, has rapidly increased. While exact pathogenetic factors are not yet well understood, overweight and obesity have been shown to be more frequent in ASD children than in controls [96]. Moreover, reduced *Bdnf* expression and excessive mTOR pathway activation have been reported [97], and *Bdnf* haploinsufficiency in human adolescents has been shown to be related to a higher score on an autism clinical rating scale [98]. The ketogenic diet has been reported to be effective in ameliorating symptoms in a mouse model [99] and in children [100]; however, to date, there are no clinical data about IF role in this setting.

Regarding epilepsy, IF has been recognized as a potential treatment since the earliest studies. In 1911, Guelpa and Marie published an article where they showed the positive effects of an IF schedule on 21 patients living with epilepsy [101]. This paper is today considered as a milestone in the history of diet-based therapies for neurological diseases. Finding new therapeutic options for epilepsy is crucial because, despite the availability of a wide array of drugs, nearly one-third of the patients affected still present seizures, despite optimal pharmacological therapy [102]. Nowadays, extensive evidence about the role of the ketogenic diet in epilepsy is available, but the same cannot be said of IF. In 2013, Hartman et al. evaluated retrospectively the effect of a short-course, modified time-restricted fasting (TRF) in a small group of children living with epilepsy whose manifestations were inadequately controlled by drugs and ketogenic diets. Authors noted that four out of six patients benefited from IF in terms of improved seizure control and three out of six were able to keep the regimen for at least 2 months [103]. This conclusion does not come as a surprise given the common traits between IF and ketogenic diet in terms of the effect on CNS metabolism. However, this pilot study suggests a potential additional effect of IF over ketogenic, non-time-restricted diets, strengthening the hypothesis of a potential positive role of an integrated nutritional approach in epileptic youngsters and paving the road for further investigations.

The use of IF in patients who have suffered an ischemic or traumatic CNS injury might also be reasonable. In fact, numerous findings show that IF-induced CNS metabolic changes can exert a neuroprotective action and improve neuronal trauma resilience. In rat models, BHB administration improved mitochondrial metabolism and neuronal stress resistance and reduced neuroinflammation markers [104]. Furthermore, IF has been demonstrated to improve motor function recovery in a rat model of spinal cord injury [85], and a single 24 h fast decreased brain damage and stimulated cognitive brain recovery after traumatic brain injury [105].

Further studies are needed to clarify the real clinical effects and possible clinical applications of IF.

## 4. Limitations, Future Perspectives, and Conclusions

Our review suggests that IF in CNS induces several neuronal adaptations, functional and structural, that overall enhance cellular stress resistance, synaptic plasticity, and neurogenesis. These effects are likely mostly triggered by BHB, whose blood levels increase during fasting. However, under conditions of food deprivation, other mediators may activate mechanisms that improve neuronal function and survival, namely hormones (ghrelin, GH, IGF-I) or changes in gut microbiota. Moreover, IF approaches in animal models have been found to improve cognitive function and, in some respects, several neurological disorders, including neurodegenerative diseases. Still, to date, several aspects remain unexplored, and some questions are still awaiting answers. First, molecular mechanisms induced by fasting need to be fully investigated: the complete understanding of the physiological processes that occur during food deprivation could lead to the recognition of possible targets that may mediate the beneficial effects of fasting, also for clinical applications. For example, glial cells and astrocytes could potentially play a role that is, so far, unexplored. Additionally, as BHB plays a major role in food deprivation, studies with a control group under a ketogenic diet could elucidate similarities and differences in the molecular changes induced from these two interventions. Moreover, as already reported by other authors [106], we are convinced that the main issue for the application of IF in clinical settings is the lack of guidelines that should guide, wherever possible, the choice of which IF regimen is a better fit for a particular subject, and that should indicate the types of food to consume, how to practice physical activity, and how long this eating pattern approach should last. Furthermore, a salient finding of the present literature review is that most studies in humans to date assessing the beneficial effects of IF on cognitive function have been observational, whereas a small clinical study reported no beneficial effects on cognition (Table 1). Well-controlled clinical trials that are designed to study the effects of IF on cognition compared to isocaloric diets with no time restriction would be fundamental to increase our understanding. Since IF regimes may also lead to unintentional reduction in energy intake (and/or body weight), in both proposed study designs, a supervised controlled feeding approach, such as the one recently performed by Sutton et al., will help to recognize weight-loss-independent effects of IF on cognitive function [107]. Other critical concerns arise from the limited duration and follow-up period of the studies conducted to date: trials with long-term interventions would provide more solid results and could also clarify if people can effectively maintain an IF regimen for years. This is not a secondary aspect, given that in some studies the adherence to fasting interventions has been variable and the dropout rate has been higher than in CR diets [108]. Another consideration is the issue of safety, as IF could potentially expose patients with type 2 diabetes mellitus, often overweight or obese, to an increased risk of hypoglycemia [109]. Moreover, long-term studies could provide information about the impact of IF on quality of life, mood, and behavioral changes, as food deprivation can potentially expose people to hunger, irritability, and reduced ability to concentrate [32]. Finally, in order to understand whether IF could prevent or improve outcomes in neurodegenerative diseases, randomized controlled trials in subjects at high risk of neurological disorders or at an initial stage of the disease are absolutely necessary. Thus, although despite the increasing number of data available, a lot has to be fully understood yet, and the impact of IF on brain health necessitates further investigation.

## Figures and Tables

**Table 1:** Summary of the studies on animals and humans employing IF and its effects on cognitive function *.

Reference	Population	IF Regimen	IF Duration	Effects of IF Cognitive Function
Animal Models				
Liu et al. [43]	T2D mice	ADF	28 days	Improvement in spatial memory and cognitive function
Hu et al. [41]	Vascular dementia rat model	ADF	12 weeks	Better cognitive performance
Fontán-Lozano et al. [82]	Mice	ADF	6–8 months	Improved spatial learning and memory
Halagappa et al. [83]	Alzheimer disease mice model	ADF	14 months	Better spatial memory acquisition and cognitive performance
Jeong et al. [85]	Thoracic spinal cord contusion injury rat model	ADF	3 weeks or –24 hrs before trauma to 10 weeks after	Decreased brain damage and stimulated cognitive brain recovery after injury
**Human Studies**				
BaHammam et al. [86]	8 healthy men	Ramadan	2 weeks	no effect in reaction time
Ooi et al. [87]	99 MCI Malay adults	ADF (5/2days)	36 months	Better cognitive performance
Anton et al. [88]	10 Overweight, sedentary subjects with mild to moderate functional limitations	TRF (16/8h)	4 weeks	No differences in cognitive function tests
Currenti et al. [89]	883 elderly Italians	TRF	observational	lower risk for cognitive impairment
Currenti et al. [90]	1572 Italian adults	TRF	observational	decreased risk for mental health distress

* studies assessing the effect of Ramadan on cognition apart from the study from Bahammam et al. are not reported in this table due to the presence of possible confounders (for instance on the quality of sleep). A recent review on this topic is provided in the main text.

## Data Availability

Not applicable.
